# Supplementary material for: Resolving the full spectrum of human genome variation using Linked-Reads
Source: Genome Res. 2019 Apr;29(4):635–45. doi: 10.1101/gr.234443.118 (PMC6442396; doi:10.1101/gr.234443.118)
Supplement: Supplemental Material [file supp_gr.234443.118_Supplemental_longranger-2.2.zip › longranger-2.2.tar.bz2!/lr2.2-public/lib/stan/cmdstan/stan/doc/api/html/check__finite_8hpp_source.html]

Stan: src/stan/math/error\_handling/check\_finite.hpp Source File


|  |  |
| --- | --- |
|  | Stan  2.5.0  probability, sampling & optimization |


- Main Page
- Related Pages
- Namespaces
- Classes
- Files

- File List
- File Members

All Classes Namespaces Files Functions Variables Typedefs Enumerations Enumerator Friends Macros Pages

- src
- stan
- math
- error\_handling

check\_finite.hpp

Go to the documentation of this file.

1 #ifndef STAN\_\_MATH\_\_ERROR\_HANDLING\_CHECK\_FINITE\_HPP

2 #define STAN\_\_MATH\_\_ERROR\_HANDLING\_CHECK\_FINITE\_HPP

3

4 #include <stan/math/error\_handling/dom\_err.hpp>

5 #include <stan/math/error\_handling/dom\_err\_vec.hpp>

6 #include <boost/math/special\_functions/fpclassify.hpp>

7 #include <stan/meta/traits.hpp>

8

9 namespace stan {

10  namespace math {

11

12  namespace {

13  template <typename T\_y,

14  typename T\_result,

15  bool is\_vec>

16  struct finite {

17  static bool check(const char\* function,

18  const T\_y& y,

19  const char\* name,

20  T\_result\* result) {

21  if (!(boost::math::isfinite)(y))

22  return dom\_err(function,y,name,

23  " is %1%, but must be finite!","",

24  result);

25  return true;

26  }

27  };

28

29  template <typename T\_y, typename T\_result>

30  struct finite<T\_y, T\_result, true> {

31  static bool check(const char\* function,

32  const T\_y& y,

33  const char\* name,

34  T\_result\* result) {

35  using stan::length;

36  for (size\_t n = 0; n < length(y); n++) {

37  if (!(boost::math::isfinite)(stan::get(y,n)))

38  return dom\_err\_vec(n,function,y,name,

39  " is %1%, but must be finite!","",

40  result);

41  }

42  return true;

43  }

44  };

45  }

46

51  template <typename T\_y, typename T\_result>

52  inline bool check\_finite(const char\* function,

53  const T\_y& y,

54  const char\* name,

55  T\_result\* result) {

56  return finite<T\_y,T\_result,is\_vector\_like<T\_y>::value>

57  ::check(function, y, name, result);

58  }

59  }

60 }

61 #endif

dom\_err.hpp

stan::math::dom\_err

bool dom\_err(const char \*function, const T &y, const char \*name, const char \*error\_msg, const T\_msg error\_msg2, T\_result \*result)

**Definition:** dom\_err.hpp:36

boost::math::isfinite

bool isfinite(const stan::agrad::var &v)

Checks if the given number has finite value.

**Definition:** boost\_fpclassify.hpp:39

stan::length

size\_t length(const T &)

**Definition:** traits.hpp:159

stan::math::check\_finite

bool check\_finite(const char \*function, const T\_y &y, const char \*name, T\_result \*result)

Checks if the variable y is finite.

**Definition:** check\_finite.hpp:52

stan::math::dom\_err\_vec

bool dom\_err\_vec(const size\_t i, const char \*function, const T &y, const char \*name, const char \*error\_msg, const T\_msg error\_msg2, T\_result \*result)

**Definition:** dom\_err\_vec.hpp:33

traits.hpp

dom\_err\_vec.hpp

stan::get

T get(const T &x, size\_t n)

**Definition:** traits.hpp:143


---

[ Stan Home Page ]

*© 2011–2014,
Stan Development Team.*
